# Supplementary material for: Effect of Dietary Phosphate Deprivation on Red Blood Cell Parameters of Periparturient Dairy Cows
Source: Animals (Basel). 2023 Jan 25;13(3):404. doi: 10.3390/ani13030404 (PMC9913789; doi:10.3390/ani13030404)
Supplement: Supplementary file 1 [file animals-13-00404-s001.zip › animals-2151525-Supplementary.pdf]

Supplementary Table S1. Ingredients (% of DM) and composition (g/kg of DM unless noted otherwise) of experimental feed rations of the adequate P (AP) and low P (LP) treatment ante-partum and post-partum

Item	AP ante-partum	LP ante-partum	post-partum
Ingredient			
Corn silage	38.3	38.2	25.5
Pressed Pulp	25.7	25.6	14.0
Hay	8.9	8.9	5.4
Straw	8.5	8.5	0
Grass silage	0	0	19.5
Concentrate pellets[1]	18.6	18.8	0
Concentrate feed[2]	0	0	35.6
Chemical analysis			
DM (% as fed)	36.6	36.5	34.7
NE_L (MJ/kg of DM)	5.72	5.68	6.86
aNDF	462	451	377
ADF	272	251	212
TDN	670	680	700
Crude Protein	120	117	168
Ca	7.2	7.6	8.9
K	10.9	11.1	15.3
P	3.0	1.6	4.6
Mg	3.7	3.5	2.6
S	4.1	2.9	3.0
Na	2.2	2.3	2.1
Cl	4.5	4.7	4.8
DCAD (mEq/kg of DM)	+50	+40	+160

Values for the experimental rations are the mean values of 9 analyses conducted throughout the study period

[1]Pelleted concentrate containing P (AP 11.0 g/kg and LP 1.5g/kg), Ca (AP 6.5 g/kg and LP 7.0 g/kg), $NH_4H_2PO_4$ (AP 31.3 g/kg and LP 0.0 g/kg), Urea (AP 19.8 g/kg and LP 33.6 g/kg), identical for AP and LP: Mg (13.0 g/kg), Na (12.0 g/kg), Cu (0.09 g/kg), Co (3.84 mg/kg), Mn (0.27 g/kg), Zn (0.41 g/kg), Se (2.04 mg/kg), vitamin A (39000 IU/kg), vitamin D_3 (6100 IU/kg), vitamin E (0.407 g/kg).

[2]Concentrate feed containing P (6.1 g/kg), Ca (6.5 g/kg), Mg (13.0 g/kg), Na (3.5 g/kg), Cu (0.03 g/kg), Co (0.55 mg/kg), Mn (0.13 g/kg), Zn (0.17 g/kg), Se (0.93 mg/kg), vitamin A (13800 IU/kg), vitamin D_3 (2160 IU/kg), vitamin E (0.07 g/kg)
